# Genome sequence of a pathogenic *Corynebacterium ulcerans* strain isolated from a wild boar with necrotizing lymphadenitis

**DOI:** 10.1186/s13104-019-4704-3

**Published:** 2019-10-25

**Authors:** Anne Busch, Jens Möller, Andreas Burkovski, Helmut Hotzel

**Affiliations:** 1 Institut für Bakterielle Infektionen und Zoonosen, Friedrich-Loeffler-Institut, Bundesforschungsinstitut für Tiergesundheit, Jena, Germany; 2 Friedrich-Alexander-Universität Erlangen-Nürnberg, Erlangen, Germany

**Keywords:** Emerging pathogen, Genomics, Toxigenic corynebacteria, Zoonotic transmission

## Abstract

**Objectives:**

*Corynebacterium ulcerans* can colonize a wide variety of animals and also humans are infected, typically by zoonotic transmission. Symptoms range from skin ulcers or systemic infections to diphtheria-like illness. In contrast, *Corynebacterium pseudotuberculosis* is widely distributed among herds of sheep, goats and other farm animals, where it causes high economic losses due to caseous lymphadenitis. Here we describe the genome sequence of an atypical *C. ulcerans* strain isolated from a wild boar with necrotizing lymphadenitis. This strain has similarities to *C. pseudotuberculosis.*

**Data description:**

Genome sequence data of *C.* *ulcerans* isolate W25 were generated, analyzed and taxonomical relationship to other *Corynebacterium* species as well as growth properties of the isolate were characterized. The genome of *C.* *ulcerans* W25 comprises 2,550,924 bp with a G+C content of 54.41% and a total of 2376 genes.

## Objective

The genus *Corynebacterium* (*C.*) comprises more than one hundred species with about half of these isolated from human and animal material [1, 2]. While most species are only rarely causing disease, others are connected to severe infections. This is especially true for the group of toxigenic corynebacteria [3], i.e. *C.* *diphtheriae*, *C.* *ulcerans* and *C.* *pseudotuberculosis*. *C.* *diphtheriae* is almost exclusively restricted to humans and is the etiological agent of diphtheria. In contrast, *C. ulcerans* can colonize a wide variety of animals and also humans are infected, typically by zoonotic transmission. In the case of human infections, skin ulcers and diphtheria-like illnesses are most common, besides cases of systemic infections. *C.* *pseudotuberculosis* is widely distributed among herds of sheep, goats and other farm animals, where it causes high economic losses due to caseous lymphadenitis. Human infections with this species are extremely rare and restricted to persons with close animal contact.

Here, we describe the genome sequence of an atypical *C. ulcerans* strain isolated from a wild boar with necrotizing lymphadenitis. Sequence data of *C. ulcerans* isolate W25 were generated and assembled and taxonomical relationship to other *Corynebacterium* species was characterized. Since only a very limited number of *C.* *ulcerans* whole genome sequences are available, the data may be valuable for taxonomical investigations and the prediction of pathogenicity based on genome mining approaches [4–7].

## Data description

The data represent genome sequence information of *C.* *ulcerans* strain W25, isolated from a hunted wild boar (*Sus* *scrofa*). The chromosomal DNA of *C.* *ulcerans* W25 was sequenced using Illumina MiSeq and deposited at DDBJ/ENA/GenBank under the accession VFEM00000000 (Table 1), which is also the version described in this paper. The genome assembly consisted of 13 contigs with an estimated total size of 2,550,924 bp and a G+C content of 54.41%. A 50-fold coverage of the genome sequence was obtained with an N50 of 328,900 bp. A total of 2376 genes with 2013 coding genes, 304 pseudogenes, and 59 RNA genes were identified. Compared to five published genome sequences [8, 9] no significant variations in respect to sequence length, the number of coding sequences and RNA genes was found. In contrast, the G+C content of the genomic DNA of the W25 strain is with 54.4%, 1.0% to 1.1% higher than in other *C.* *ulcerans* strains (see Data set 2, Table 1).

**Table 1:** Overview of data files/data sets

Label	Name of data file/data set	File types (file extension)	Data repository and identifier (DOI or accession number)
The whole-genome shotgun sequencing project	The whole-genome shotgun sequencing project	No file type	https://www.ncbi.nlm.nih.gov/nuccore/VFEM00000000.1/ [15]
Sequence read archive	SRX6047294: Genome sequence of a pathogenic *Corynebacterium ulcerans* strain isolated form a wild boar with necrotizing lymphadenitis	1 ILLUMINA (Illumina MiSeq) run: 700,493 spots, 333.3 M bases, 194.7 Mb downloads	https://www.ncbi.nlm.nih.gov/sra/SRX6047294 [15]
Assembly data	ASM637053v1	fasta	https://www.ncbi.nlm.nih.gov/assembly/GCA_006370535.1 [15]
BioSample	*Corynebacterium ulcerans* strain W25	No file type	SAMN12027930: https://www.ncbi.nlm.nih.gov/biosample/SAMN12027930/ [15]
BioProject	*Corynebacterium ulcerans* strain W25 Genome sequencing and assembly	No file type	PRJNA548458: https://www.ncbi.nlm.nih.gov/bioproject/548458 [15]
Supplemental material W25	Supplemental material W25 (Data_set_1_supplemental_material_W25)	Pdf	10.6084/m9.figshare.8397245
Table S1	Table S1 (Data_set_2_table_S1)	Pdf	10.6084/m9.figshare.8397320

The data set provided includes a PDF file (Data set 1) containing two images of the growth behavior of the isolate as well as a phylogenetic tree of corynebacteria reflecting an atypical phenotype of *C.* *ulcerans* W25 by its close taxonomical relationship to *C. pseudotuberculosis* (Table 1).

## Methodology

### Growth of bacteria

*Corynebacterium ulcerans* isolate W25 was isolated from a hunted wild boar and propagated as a pure culture on Columbia Blood Agar (CBA) plates. On this solid medium, the bacteria had a waxy appearance and showed no hemolysis (Data set 1, Table 1). For subsequent experiments, *C.* *ulcerans* strains were grown in Brain Heart Infusion (BHI) containing 10% fetal bovine serum (FBS) and 0.05% Tween 80.

### Genome sequencing

After 72 h of cultivation in BHI DNA was prepared using QIAGEN Genomic-tips 20/G and a QIAGEN Genomic DNA Buffer Set (Qiagen, Hilden, Germany). The DNA quality was examined by using a Qubit 2.0 fluorometer (Life Technologies, Darmstadt, Germany) and by agarose gel electrophoresis. Nextera XT Library Preparation Kit library according to the manufacturer’s instruction. Sequencing was done with an Illumina MiSeq run 2 × 300 bp. Quality was assessed and assembled with SPAdes v. 3.11.1 (with the additional command-careful) [10] was used and for annotation the Prokka annotation pipeline 1.12-beta in standard settings [11] as described before [12]. The mean coverage was 236 reads with a standard deviation of 71 reads. Mapped to the *Corynebacterium ulcerans* BR-AD22 77.4% was covered with a mean coverage of 57 reads.

### Data analysis

To assess the phylogenetic classification of assorted *Corynebacterium* species PhyloPhlAn was used with the annotation files resulting from Prokka. The analysis was performed with standard-setting on all samples and visualized with Dendroscope as described before [12–14].

## Limitations

The data represent the first characterization of genome sequence data of a newly isolated *C. ulcerans* strain. For further analyses, it may be necessary to close existing gaps and improve and cure the current annotation. For example, long-read sequencing (PacBio or MinIon) could generate in a hybrid assembly a more conclusive picture of the genome structure and possibly regulative aspects of protein expression.

## Data Availability

The data described in this Data note can be freely and openly accessed on DDBJ/ENA/GenBank under the accession https://www.ncbi.nlm.nih.gov/nuccore/VFEM00000000.1/. Please see Table 1 and Reference list for details and links to the data.

## Abbreviations

BHI: brain heart infusion
CBA: Columbia Blood Agar
FBS: fetal bovine serum
bp: base pair(s)
